# Supplementary material for: Exceptional longevity of mammalian ovarian and oocyte macromolecules throughout the reproductive lifespan
Source: eLife. 2024 Oct 31;13:RP93172. doi: 10.7554/eLife.93172 (PMC11527430; doi:10.7554/eLife.93172)

Figure 3—figure supplement 1A – source data 2

4-16% Tris-glycine gel, oocyte proteins lysate

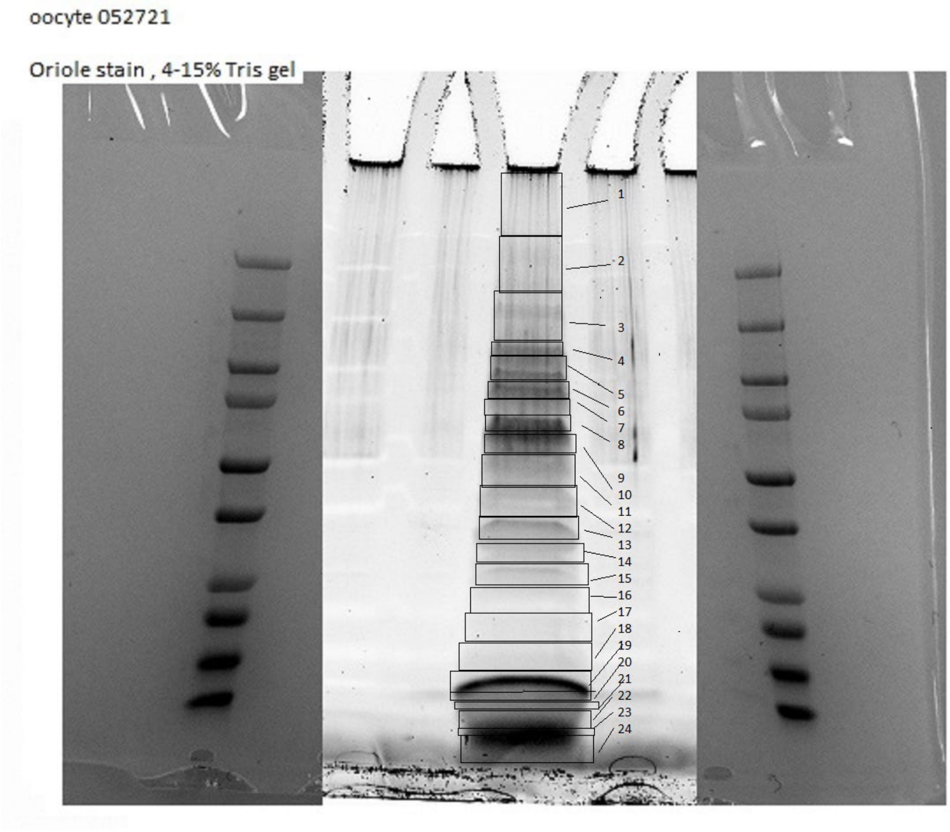

Supplement: Figure 3—figure supplement 1—source data 2. [file elife-93172-fig3-figsupp1-data2.zip › Figure 3 source 2.pdf]
